# Maximum Correntropy Based Unscented Particle Filter for Cooperative Navigation with Heavy-Tailed Measurement Noises

**DOI:** 10.3390/s18103183

**Published:** 2018-09-20

**Authors:** Ying Fan, Yonggang Zhang, Guoqing Wang, Xiaoyu Wang, Ning Li

**Affiliations:** Department of Automation, Harbin Engineering University, Nantong Road No.145, Harbin 150001, China; fine@hrbeu.edu.cn (Y.F.); wangguoqing2014@hrbeu.edu.cn (G.W.); wangxiaoyu666@hrbeu.edu.cn (X.W.); ningli@hrbeu.edu.cn (N.L.)

**Keywords:** autonomous underwater vehicle (AUV), cooperative navigation, maximum correntropy criterion, unscented particle filter, measurement outliers, KLD-resampling

## Abstract

In this paper, a novel robust particle filter is proposed to address the measurement outliers occurring in the multiple autonomous underwater vehicles (AUVs) based cooperative navigation (CN). As compared with the classic unscented particle filter (UPF) based on Gaussian assumption of measurement noise, the proposed robust particle filter based on the maximum correntropy criterion (MCC) exhibits better robustness against heavy-tailed measurement noises that are often induced by measurement outliers in CN systems. Furthermore, the proposed robust particle filter is computationally much more efficient than existing robust UPF due to the use of a Kullback-Leibler distance-resampling to adjust the number of particles online. Experimental results based on actual lake trial show that the proposed maximum correntropy based unscented particle filter (MCUPF) has better estimation accuracy than existing state-of-the-art robust filters for CN systems with heavy-tailed measurement noises, and the proposed MCUPF has lower computational complexity than existing robust particle filters.

## 1. Introduction

Accurate navigation and localization of autonomous underwater vehicles (AUVs) are paramount for AUV autonomy. However, due to the existence of rapid attenuation in electro-magnetic, radio-frequency, and global position system (GPS) signals underwater, localization of AUVs has always been an intractable problem [1,2,3]. Inertial navigation system (INS) equipped high-accuracy inertial sensors is always employed in AUV navigation. However, the error of AUVs equipped with INS is accumulated over time and causes the localization error increase unboundedly [4,5]. With the aid of GPS signal, which can be obtained close to surface, the localization error can be alleviated, but it’s hard to realize in deep-water. Another method is to use acoustic baseline in AUVs, whose localization error is bounded [6,7,8]. Unfortunately, there is a high cost in equipment and limited working range for using static beacons. In this case, cooperative navigation (CN) based on acoustic range observation has been proposed [6,9,10,11]. High accuracy inertial sensors are installed in a small amount of AUVs in a CN system, which can assist the other AUVs that equipped with low precision dead-reckoning (DR) by using acoustic modems [5,11,12]. The CN of AUVs enables navigation information to be shared among each AUV, which makes the using scope more flexible and improves work efficiency of AUVs. For cooperative localization of AUVs, position estimation is of great importance and filtering technique based on state-space model has always been employed to solve this problem [4,9]. 

Particle filter (PF) is a popular method to address the state estimation problem in CN systems, in which the posterior probability density function (PDF) is approximated by a set of weighted particles based on a sequential Monte Carlo (MC) method [13,14,15]. A key component for PF is to select an appropriate importance PDF. In the conventional PF, the importance PDF is usually chosen as the prior state transition PDF for easy sampling and weight calculation [14,16]. However, state transition PDF is not the best choice for importance PDF since it does not include the latest measurement information. To solve this problem, an extended particle filter (EPF) has been proposed, where the extended Kalman filter (EKF) is employed to generate importance PDF [16,17,18]. The EPF contains not only prior information but also the latest measurement information so that it can match the optimal importance PDF (that is the true posterior PDF) better than conventional PF [14,17,18]. Based on a similar approach, an unscented particle filter (UPF) has also been proposed by utilizing the unscented Kalman filter (UKF) to generate the importance PDF. As compared with the EPF, the UPF can achieve better estimation accuracy [8,17,19,20]. 

**Figure 1:**
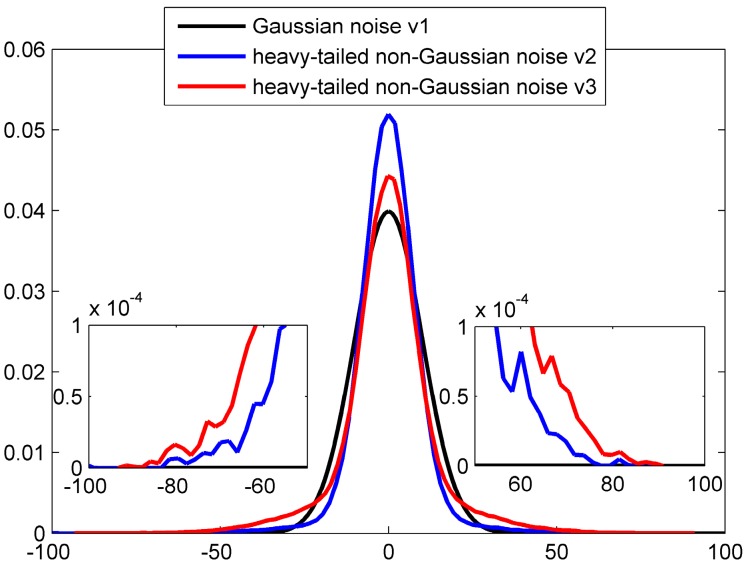
Gaussian distribution and heavy tailed non-Gaussian distribution induced by outlier interference.

Both EKF and UKF require the Gaussian assumption of measurement noise. However, there may be outliers of velocity and range in practical cooperative localization of AUVs, which results in a heavy-tailed non-Gaussian measurement noise [4,6,21]. Physically misalignment of body framework in Doppler velocity log (DVL) or water lock may induce outlier measurements of velocity [22]. Multiple acoustic propagation paths between receivers and source, which are caused by the reflection of sound wave that are caused by the changes of sound speed with depth and reflections off the sea bed and surface, may induce the outlier in range measurements [23]. In fact, such outliers lead to a bias from Gaussian distribution and can be modeled by a distribution that has heavier tails [21]. Outlier corrupted noises are generated according to:

(1) $$v\sim \{\begin{array}{cc} N\left(0,R\right) & w.p.\;(1-\varepsilon )\\ N(0,10R) & w.p.\;\varepsilon \end{array}$$

where N(β,Σ) represents the Gaussian distribution with mean value β and covariance Σ; ε is a perturbing parameter of outliers contamination. The PDF of Gaussian noise $v_{1}$ ($v_{1}\sim N\left(0,R\right)$, R=10), heavy-tailed non-Gaussian noises $v_{2}$ (generated based on Equation (1) with ε=0.1) and heavy-tailed non-Gaussian noises $v_{3}$ (generated based on Equation (1) with ε=0.3) are shown in Figure 1. It can be seen that heavy-tailed non-Gaussian noise $v_{2}$ has more heavy tail than $v_{3}$ because that $v_{2}$ contains more outliers. 

As a result, the estimation performance of EPF and UPF will degrade severely for such heavy-tailed non-Gaussian measurement noises. To address heavy-tailed measurement noises, many methods have been proposed, such as the Huber-based nonlinear Kalman filters (HNKFs) [6,16,24,25,26,27,28]. The HNKF is a kind of commonly used robust filter but its influence function doesn’t redescend, which results in deteriorating estimation performance [29]. By approximating the posterior density function (PDF) as a Student’s t distribution, a robust Student’s t based nonlinear filter (RSTNF) has been proposed [30,31,32,33]. However, such Student’s t approximation may be unreasonable in some engineering applications with slightly heavy-tailed measurement noises, which may result in deteriorating filtering performance [30]. To solve this problem, a novel robust Student’s t based Kalman filter (RSTKF) has been proposed based on variational-Bayesian (VB) method [26]. In the RSTKF, the posterior PDF is approximated as a Gaussian distribution. The RSTKF has good estimation accuracy but suffers from intensive computation. The correntropy, which is a local similarity measure in information theoretic, has been gaining more and more attention. The correntropy can capture higher-order statistical information of data directly not only the usual second-order statistical information, which has the potential to achieve better estimation performance [31,32]. Based on the maximum correntropy criterion (MCC), several robust filters including maximum correntropy Kalman filter (MCKF) and maximum correntropy unscented Kalman filter (MCUKF) have been proposed to suppress the impulsive noises [31,32,33,34,35].

In this paper, to further improve the positioning accuracy of UPF under non-Gaussian heavy-tailed measurement noise in CN of AUVs, a new maximum correntropy based unscented particle filter (MCUPF) is proposed, which modifies the update process of importance sampling of UPF. The proposed MCUPF exhibits good robustness to deviations from Gaussian distribution and has a recursive structure. Also, the Kullback-Leibler Divergence (KLD)-resampling is employed to further improve the computational efficiency of the proposed algorithm, in which the KLD is used to measure the approximate error of distribution represented by weighted particles to adjust the number of particle adaptively [36,37]. 

The remainder of this paper is organized as follows. In Section 2, the model of cooperative navigation and UPF algorithm are briefly reviewed. In Section 3, a new MCUPF is proposed. In Section 4, the proposed MCUPF and existing state-of-the-art robust filters are compared through an actual lake trial of AUVs, and simulation results are obtained. The conclusion is drawn in Section 5.

## 2. Problem Formulation

### 2.1. System Model

Now, we consider the model of cooperative navigation, which is based on a master-slave mode of AUVs. As the depth of AUVs can be measured accurately by pressure sensors, the three dimensional (3-D) model of CN can be transformed into two dimensional (2-D) model. The state space model of CN system based on acoustic range are described as follows [10]:

(2) $$\{\begin{array}{c} x_{k}=x_{k-1}+\Delta t\left({\hat{c}}_{k}\cos{\hat{\theta }}_{k}+{\hat{s}}_{k}\sin{\hat{\theta }}_{k}\right)+w_{x,k-1}\\ y_{k}=y_{k-1}+\Delta t\left({\hat{c}}_{k}\sin{\hat{\theta }}_{k}-{\hat{s}}_{k}\cos{\hat{\theta }}_{k}\right)+w_{y,k-1} \end{array}$$

(3) $$z_{k}=\sqrt{{\left(x_{k}-x_{k}^{r}\right)}^{2}+{\left(y_{k}-y_{k}^{r}\right)}^{2}}+v_{k}$$

where $x_{k}$ and $y_{k}$ are east and north positions of AUVs at time k, respectively; $x_{k}^{r}$ and $y_{k}^{r}$ are east and north positions of communication and navigation aid (CNA) at time k provided periodically by the acoustic modern, respectively; Δt is the sampling time, ${\hat{s}}_{k}$ and ${\hat{c}}_{k}$ are starboard and forward velocities at time k, respectively, which are provided by DVL; ${\hat{\theta }}_{k}$ is heading angle measured by compass. 

Based on Equations (2) and (3), the state-space model of CN system can be formulated as follows:

(4) $$\{\begin{array}{c} x_{k}=Fx_{k-1}+\mu_{k}+w_{k-1}\\ z_{k}=h_{k}\left(x_{k}\right)+v_{k} \end{array}$$

where $x_{k}={\left[x_{k},y_{k},\theta_{k}\right]}^{T}$ denotes state vector, $F=E_{2}$ is state transition matrix and $E_{2}$ represents the 2-D identity matrix, $w_{k-1}={\left[w_{x,k-1},w_{y,k-1}\right]}^{T}$ denotes process noise vector, $\mu_{k}$ denotes the control output and $z_{k}$ denotes relative distance between CNA and AUV. The process noise $w_{k-1}$ and measurement noise $v_{k}$ are mutually uncorrelated Gaussian white noise processes. Here, $w_{k-1}\sim N\left(0,Q_{k}\right)$ and $v_{k-1}\sim N\left(0,R_{k}\right)$, where $Q_{k-1}$ denotes process noise covariance matrix and $R_{k}$ denotes measurement noise covariance. Besides, there may be correlations between process noise $w_{x,k-1}$ and $w_{y,k-1}$ because both of them rely on the measurement errors of compass and DVL. 

### 2.2. Review of the Standard UPF Algorithm 

Before deriving the proposed MCUPF, we first briefly introduce the standard UPF. As compared with conventional PF, UPF modifies the importance PDF through the UKF to include the latest observation information, which requires to assume that $w_{k-1}$ and $v_{k}$ have Gaussian distributions. The UPF algorithm is summarized as follows [14,19,20].

Initialization: Inputs ${\hat{x}}_{0|0}^{\left(j\right)}$, ${\hat{P}}_{0|0}^{\left(j\right)}$ and the number of particles *N*. Draw *N* particles $x_{0|0}^{\left(j\right)}\;\left(j=1,\;2,\;\ldots \;,\;N\right)$ from the known prior distribution $p\left(x_{0}\right)$, with:

(5) $${\hat{x}}_{0|0}^{\left(j\right)}=E\left[x_{0|0}^{\left(j\right)}\right]$$

(6) $${\hat{P}}_{0|0}^{\left(j\right)}=E\left[\left(x_{0|0}^{\left(j\right)}-{\hat{x}}_{0|0}\right){\left(x_{0|0}^{\left(j\right)}-{\hat{x}}_{0|0}\right)}^{T}\right]$$

and the weights of all particles are set as $f_{0}^{\left(j\right)}=\frac{1}{N}$.

For k=1:T

Importance sampling through UKF:

For j=1:N

1. Time Update

Calculate the sigma points:

(7) $$X_{i,k-1|k-1}=\left\{ \begin{array}{ll} {\hat{x}}_{k-1|k-1}^{\left(j\right)}, & d_{i}=\frac{\kappa }{\lambda +\kappa },\;i=0\\ {\hat{x}}_{k-1|k-1}^{\left(j\right)}\pm \sqrt{\left(n+\lambda \right){\hat{P}}_{k-1|k-1}^{\left(j\right)}}, & d_{i}=\frac{1}{2\left(\lambda +\kappa \right)},\;i=1\;,\;2,\;\ldots \;,\;2n+1 \end{array}\right.$$

where $d_{i}$ are the weights of sigma points and n is the dimension of state vector; the free parameter of UKF is set as λ=3−n.

Calculate the propagated sigma points:

(8) $$X_{i,\;k|k-1}^{(j)*}=FX_{i,k-1|k-1}$$

Calculate the one-step prediction:

(9) $${\hat{x}}_{k|k-1}^{\left(j\right)}=\sum_{i=0}^{2n+1}d_{i}X_{i,\;k|k-1}^{(j)*}$$

Calculate the one-step prediction error covariance matrix:

(10) $${\hat{P}}_{k|k-1}^{\left(j\right)}=\sum_{i=0}^{2n+1}d_{i}\left(X_{i,\;k|k-1}^{\left(j\right)*}-{\hat{x}}_{k|k-1}^{\left(j\right)}\right){\left(X_{i,\;k|k-1}^{\left(j\right)*}-{\hat{x}}_{k|k-1}^{\left(j\right)}\right)}^{T}+Q_{k-1}\;$$

2. Measurement Update

Calculate the sigma points:

(11) $$X_{i,k|k-1}=\left\{ \begin{array}{lll} {\hat{x}}_{k|k-1}^{\left(j\right)}, & d_{i}=\frac{\kappa }{n+\kappa } & i=0\\ {\hat{x}}_{k|k-1}^{\left(j\right)}\pm \sqrt{\left(n+\kappa \right){\hat{P}}_{k|k-1}^{\left(j\right)}}, & d_{i}=\frac{1}{2\left(n+\kappa \right)} & i=1\;,\;2,\;\ldots \;,\;2n+1 \end{array}\right.$$

Calculate the propagated sigma points (i=1 , 2, … , 2n+1):

(12) $$Z_{i,\;k|k-1}^{(j)*}=h\left(X_{i,\;k|k-1}\right)$$

Calculate the predicted measurement vector (i=1 , 2, … , 2n+1):

(13) $${\hat{z}}_{k|k-1}^{\left(j\right)}=\sum_{i=0}^{2n+1}d_{i}Z_{i,\;k|k-1}^{(j)*}$$

Calculate the innovation covariance matrix, cross-covariance matrix and Kalman gain (i=1 , 2, … , 2n+1), respectively.

(14) $$P_{zz,\;k|k-1}^{\left(j\right)}=\sum_{i=0}^{2n+1}d_{i}\left(Z_{i,\;k|k-1}^{(j)*}-{\hat{z}}_{k|k-1}^{\left(j\right)}\right){\left(Z_{i,\;k|k-1}^{(j)*}-{\hat{z}}_{k|k-1}^{\left(j\right)}\right)}^{T}+R_{k}$$

(15) $$P_{xz,\;k|k-1}^{\left(j\right)}=\sum_{i=0}^{2n+1}d_{i}\left(X_{i,\;k|k-1}^{(j)*}-{\hat{x}}_{k|k-1}^{\left(j\right)}\right){\left(Z_{i,\;k|k-1}^{(j)*}-{\hat{z}}_{k|k-1}^{\left(j\right)}\right)}^{T}$$

(16) $$W_{k}^{\left(j\right)}=P_{xz,\;k|k-1}^{\left(j\right)}{\left(P_{zz,\;k|k-1}^{\left(j\right)}\right)}^{-1}$$

Calculate the state estimation of the *j*-th particle:

(17) $${\hat{x}}_{k|k}^{\left(j\right)}={\hat{x}}_{k|k-1}^{\left(j\right)}+W_{k}^{\left(j\right)}\left(z_{k}-{\hat{z}}_{k|k-1}^{\left(j\right)}\right)$$

Calculate the estimation error covariance matrix of the *j*-th particle:

(18) $${\hat{P}}_{k|k}^{\left(j\right)}={\hat{P}}_{k|k-1}^{\left(j\right)}-W_{k}^{\left(j\right)}P_{zz,k|k-1}^{\left(j\right)}{\left(W_{k}^{\left(j\right)}\right)}^{T}$$

3. Sample from the importance PDF:

(19) $$x_{k|k}^{\left(j\right)}\text{\textasciitilde{}}q\left(x_{k}|X_{0:k-1}^{j},Z_{1:k}\right)=N\left({\hat{x}}_{k|k}^{\left(j\right)},{\hat{P}}_{k|k}^{\left(j\right)}\right)$$

4. Calculate weight of each particle:

(20) $$f_{k}^{\left(j\right)}=f_{k-1}^{\left(j\right)}\frac{p\left(z_{k}|x_{k}^{\left(j\right)}\right)p\left(x_{k}^{\left(j\right)}|x_{k-1}^{\left(j\right)}\right)}{q\left(x_{k}^{\left(j\right)}|x_{k-1}^{\left(j\right)},z_{k}\right)}$$

5. Normalize the weights:

(21) $$f_{k}^{*(j)}=\frac{f_{k}^{\left(j\right)}}{\sum_{j=1}^{N}f_{k}^{\left(j\right)}}$$

Resampling: Multiply particles with high weights and suppress particles with low weights to generate a new particle set ${\tilde{x}}_{k|k}^{\left(j\right)}\;\left(j=1,\;2,\;\ldots \;,\;N\right)$, which distributes according to $P\left(x_{0:k}|z_{1:k}\right)$ approximately.

(22) $$\left[\{{{\tilde{x}}_{k|k}^{\left(j\right)},{\tilde{f}}_{k}^{\left(j\right)}=\frac{1}{N}\}}_{j=1}^{N}\right]=\mathrm{Resampling}\left[\{{x_{k|k}^{\left(j\right)},f_{k}^{*(j)}\}}_{j=1}^{N}\right]$$

State Estimation:

(23) $${\hat{x}}_{k|k}=\sum_{j=1}^{N}{\tilde{f}}_{k}^{\left(j\right)}{\tilde{x}}_{k|k}^{\left(j\right)}$$

(24) $${\hat{P}}_{k|k}=\sum_{j=1}^{N}{\tilde{f}}_{k}^{\left(j\right)}\left({\tilde{x}}_{k|k}^{\left(j\right)}-{\hat{x}}_{k|k}\right){\left({\tilde{x}}_{k|k}^{\left(j\right)}-{\hat{x}}_{k|k}\right)}^{T}$$

The standard UPF is summarized in Table 1.

## 3. Derivation of MCUPF

Although the UPF utilizes the latest measurement information, it can’t resist the measurement outliers due to the Gaussian assumption of measurement noise. Next, MCC is firstly used to robustify the UPF in importance sampling process, and then the KLD-resampling method is used to adjust the particle number online without decreasing the estimation accuracy.

### 3.1. Brief Introduction of MCC 

Correntropy is a measure of similarity between two random variables X and Y, which is described as:

(25) $$V\left(X,Y\right)=E\left[\kappa \left(X,Y\right)\right]=\int \int \kappa \left(x,y\right)dF_{XY}\left(x,y\right)$$

where E(⋅) is the expectation operator, κ(⋅) is a shift-invariant Mercer kernel, and $F_{XY}\left(x,y\right)$ denotes the joint distribution function of X and Y. In most cases, the joint distribution $F_{XY}\left(x,y\right)$ is unknown and the only available data is a limited number of samples $\left\{x_{i},y_{i}|i=1,\ldots ,N\right\}$ from $F_{XY}\left(x,y\right)$. Thus, based on the known data, the correntropy can be evaluated as follows:

(26) $$\hat{V}\left(X,Y\right)=\frac{1}{N}\sum_{i=1}^{N}\kappa \left(x_{i},y_{i}\right).$$

The most common used Gaussian kernel is defined as:

(27) $$\kappa \left(x_{i},y_{i}\right)=G_{\sigma }\left(e_{i}\right)=\exp\left(-\frac{{\left(e_{i}\right)}^{2}}{2\sigma^{2}}\right).$$

where $e_{i}=x_{i}-y_{i}$ and σ>0 is the kernel bandwidth. In this case, the cost function of MCC can be written as:

(28) $$J_{MCC}=\frac{1}{N}\sum_{i=1}^{N}G_{\sigma }\left(e_{i}\right).$$

Assume that the vector $\hat{B}$ is the optimal solution based on MCC, the optimal solution can be obtained by maximizing the correntropy between desired signal $y_{i}$ and filter output $x_{i}$:

(29) $$\hat{B}=\underset{B\in \;\Omega }{\arg\;\max}\frac{1}{N}\sum_{i=1}^{N}G_{\sigma }\left(e_{i}\right)$$

where Ω represents the feasible solution set of $\hat{B}$. 

That is the maximum correntropy criterion, which can be incorporated in UPF to enhance robustness in non-Gaussian measurement noise.

### 3.2. Robustify the UPF

Considering that the correntropy is robust to outliers and can suppress negative impact of impulsive noises, MCC is incorporated into UKF to improve importance sampling in UPF.

Firstly, for measurement equation:

(30) $$z_{k}=h_{k}\left(x_{k}\right)+v_{k}$$

decomposing the measurement noise covariance $R_{k}$:

(31) $$R_{k}=a^{2}$$

Exploiting (31), Equation (30) can be further written as:

(32) $$C_{k}=g_{k}\left(x_{k}\right)+\zeta_{k}$$

with:

(33) $$C_{k}=\frac{1}{a}\cdot Z_{k}$$

(34) $$g_{k}\left(x_{k}\right)=\frac{1}{a}\cdot h_{k}\left(x_{k}\right)$$

(35) $$\zeta_{k}=\frac{1}{a}\cdot v_{k}.$$

The main idea of MCC is to maximize the correntropy between $C_{k}$ and $g_{k}\left(x_{k}\right)$ to obtain a minimized error $\zeta_{k,\;\min}$, i.e.:

(36) $${\hat{x}}_{k|k}=\underset{x_{k}}{\arg\;\min}\left({\left\|x_{k}-{\hat{x}}_{k|k-1}\right\|}_{V_{k}}^{2}+\left(G_{\sigma }\left(0\right)-G_{\sigma }\left(\xi_{k,i}\right)\right)\right)$$

where ${\left\|\cdot \right\|}^{2}$ represents $l_{2}$-*norm* of vector, $V_{k}$ denotes the inverse matrix of one-step predicted covariance matrix $P_{k|k-1}$ and $\xi_{k,i}$ denotes the *i*-th component of $\left(C_{k}-g_{k}\left(x_{k}\right)\right)$. $G_{\sigma }\left(\cdot \right)$ is Gaussian kernel which is defined as follows [34]:

(37) $$G_{\sigma }\left(e_{i}\right)=\exp\left(-\frac{{e_{i}}^{2}}{2\sigma^{2}}\right).$$

Considering only one fixed point iteration, the Equation (36) can be solved by using the modified measurement covariance matrix ${\tilde{R}}_{k}$ in the update process as [34,35]:

(38) $${\tilde{R}}_{k}=R_{k}/C_{k}.$$

Therefore, based on MCC, robust UPF can be obtained by modifying update process of importance sampling of UPF. The proposed robust UPF is summarized in Table 2.

**Remark** **1.** *The kernel bandwidth*σ*is an important parameter of robust UPF, which has significant effects on the estimation performance. In general, the algorithm will exhibit better robustness to outliers with a small*σ*. However, if*σ* is too small, it results in degraded performance and even diverge [33]. Furthermore, robust UPF is similar to UPF for a large *σ*, and particularly robust UPF will reduce to classic UPF when*σ→∞.

### 3.3. Modified Resampling Process

Based on the strong laws of large numbers, better estimation accuracy can be obtained when more particles are adopted [14]. Unfortunately, the greater the number of particles, the heavier the computational cost that is required. Therefore, it’s necessary to investigate some methods to improve the computational efficiency of PF. In the proposed MCUPF, the number of particles is adaptively adjusted online based on the KLD-resampling method, where KLD is used to determine the number of particles required for next iteration. 

To ensure that the KLD between the posterior distribution before resampling and the posterior distribution after resampling is less than and equal to a pre-given error, the required number $N_{KLD}$ of particles can be approximated as follows [36]:

(40) $$N_{KLD}=\frac{k-1}{2\varepsilon }{\{1-\frac{2}{9(k-1)}+\sqrt{\frac{2}{9(k-1)}}z_{1-\delta }\}}^{3}$$

where k is the number of bins with support and $z_{1-\delta }$ is the upper quartile of the standard Gaussian distribution. For a specific value of δ, the corresponding value of $z_{1-\delta }$ can be obtained from the normal distribution table. 

**Figure 2:**
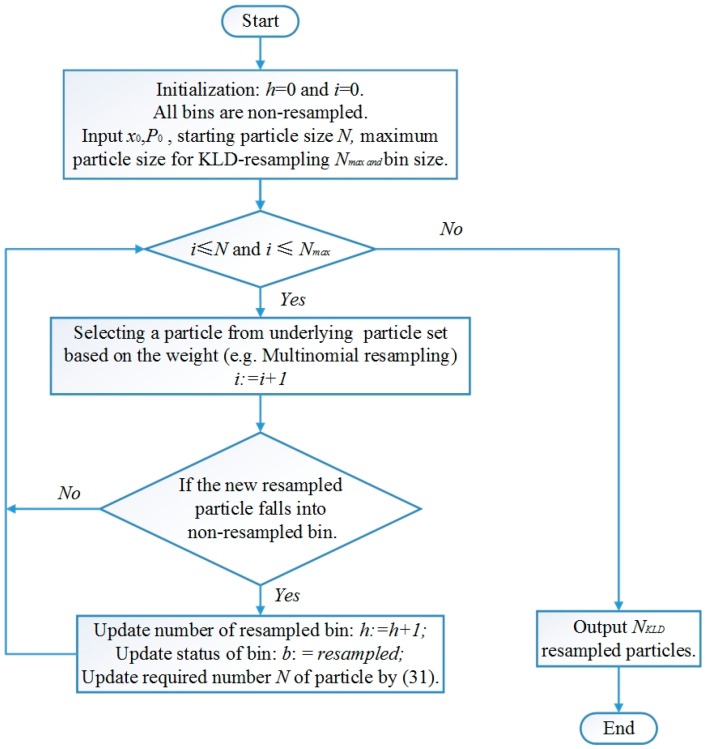
Procedure of Kullback-Leibler Divergence (KLD)-resampling.

KLD-resampling is composed of two parts as represented in Figure 2. Firstly, reselect particles based on their weights one by one until the required number $N_{KLD}$ is satisfied. Then, $N_{KLD}$ and bin size k are updated when a new sample is resampled [36].

### 3.4. The Proposed MCUPF

The proposed robust UPF is highly inefficient as a great deal of particles used for estimation. For making up the defect, the proposed MCUPF replace the resampling process with KLD-resampling, which can adjust the number of particles over time to determine the minimum amount of particles required to guarantee the estimation quality and reduce computing effort. The MCUPF, which is obtained based on MCC and KLD-resampling, is summarized in Table 3. 

It can be seen from Table 3 that the importance PDF of the proposed MCUPF is generated by the robust UKF, which includes the latest observation information and can resist to measurement outliers. In addition, according to the KLD-resampling, the sample number of the proposed MCUPF can be adaptively adjusted in real time to improve the computational efficiency.

## 4. Lake-Water Filed Trial 

**Figure 3:**
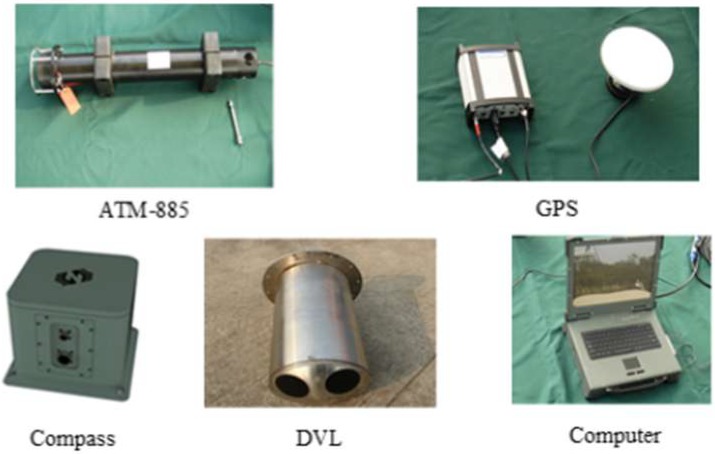
Sensors and computer employed in this test.

**Figure 4:**
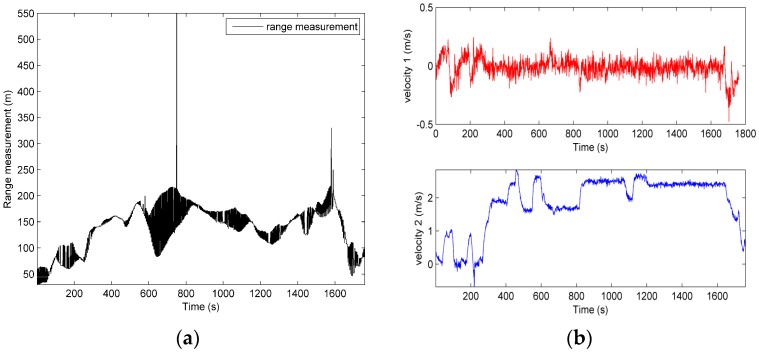
(**a**) Range measurement; (**b**) Velocity measurement.

The effectiveness of the proposed MCUPF is verified by employing the post processed date collected in an actual lake trial of AUVs. The filed trial was conducted in September 2014 in Lake Thai whose depth in most part varies seven to sixteen feet. In this test, two survey vessels acted as surface leaders known as CNAs, and only a single vessel surveyed as slaver AUV. All three vessels were equipped with an underwater acoustic modem ATM-885, and vessels can broadcast information to each other. For providing accurate position and time information to slaver AUV, the GPS OEMV-2RT-2 were installed in two CNAs. In addition, the DVL DS-99 for acquiring velocity information and a magnetic compass H/H HZ001 for obtaining a heading were also equipped on AUV. Figure 3 represents sensors and computer employed in this test and performance parameters and size of sensors used in this test are listed in Table 4 and Table 5, respectively. In this lake trial, acoustic data packets were sent from two leader CNAs to slaver AUV every 5 s. Range information and velocity information observed in this trial are presented in Figure 4 (velocity 1 and velocity 2 represent starboard velocity and forward velocity, respectively). 

**Figure 5:**
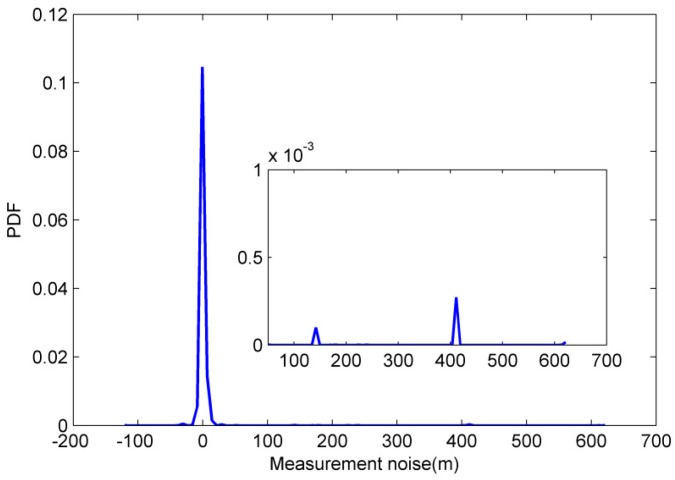
Probability density function (PDF) of measurement noise.

To further illustrate effect of outlier measurement on measurement noise, the measurement noise are obtained as follows:

(44) $${\hat{v}}_{k}=z_{k}-\sqrt{{\left({\hat{x}}_{k}-x_{k}^{r}\right)}^{2}+{\left({\hat{y}}_{k}-y_{k}^{r}\right)}^{2}}$$

where $\left({\hat{x}}_{k},{\hat{y}}_{k}\right)$ is the position of AUV at time *k* provided by GPS and ${\hat{v}}_{k}$ is approximate measurement noise value. The approximate measurement noise values can be obtained based on Equation (44) and PDF of measurement noise are shown in Figure 5. It can be seen from Figure 5 that the measurement noise have heavy-tailed distribution and Gaussian distribution can’t fit to the measurement noise value. Therefore, it’s essential to apply a robust filter algorithm to CN of AUVs.

**Figure 6:**
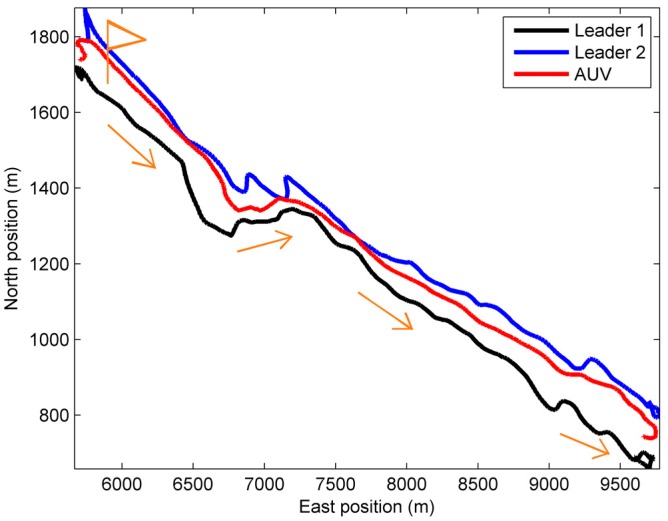
True trajectory of two leaders and a slaver autonomous underwater vehicles (AUVs).

The true north position and east position of two leaders and a slave AUV are presented in Figure 6, which are obtained using the information provided by GPSs installed on these three vessels. It’s clear to see from Figure 6 that AUV was between two leaders, and the observability of AUV can be improved by this way.

The performance of each filter is measured by position error (PE) and averaged position error (APE):

(45) $$\mathrm{PE}\left(k\right)=\sqrt{{\left({\hat{x}}_{k}-{\hat{x}}_{k|k}\right)}^{2}+{\left({\hat{y}}_{k}-{\hat{y}}_{k|k}\right)}^{2}}$$

(46) $$\mathrm{APE}=\frac{1}{T}\sum_{k=1}^{T}\sqrt{{\left({\hat{x}}_{k}-{\hat{x}}_{k|k}\right)}^{2}+{\left({\hat{y}}_{k}-{\hat{y}}_{k|k}\right)}^{2}}$$

where *T* = 1760 s is the total experimental time, $\left({\hat{x}}_{k},\;{\hat{y}}_{k}\right)$, $\left({\hat{x}}_{k|k},{\hat{y}}_{k|k}\right)$ are reference position provided by GPS and estimated position at time k, respectively. The proposed MCUPF and existing state-of-the-art filters are coded with MATLAB and the simulations are run on a computer with Intel Pentium CPU at 2.90 GHz with 2.00 GB memory.

### 4.1. Comparisons of Different Filtering Methods 

To save computation cost, the bins in KLD-resampling is usually divided in new state space which has a lower dimension than the origin state vector. The bin size is selected in terms of process noise covariance [36]. Following the advice of [36], bins in KLD-resampling of the proposed MCUPF is divided in 2-D (bin size is [10,10]). The process noise covariance matrix is $Q_{k}=diag\left(\left[{\left(10m\right)}^{2},\;{\left(10m\right)}^{2}\right]\right)$ and measurement noise covariance is R=0.1.

The existing PF [14], UPF [14], Huber unscented particle filter (HRUPF) [24], MCUKF [34], iterated VB based cubature Kalman filter (IVBCKF) [21], and the proposed MCUPF are compared, where the simple random resampling is employed in PF and UPF. In order to obtain a better performance of MCUKF, the kernel bandwidth in MCUKF and MCUPF are set as σ=15 following the advice of [34,35]. Besides, the turning parameter and iterated times in HRUPF are commonly used γ=1.345 and m=3 [24,27], respectively. In IVBCKF, conjugate prior distributions for scale matrices of prior state and likelihood are inverse Wishart distribution. $u_{k}$ and $t_{k}$ denote degrees of freedom (dof) parameters of these two different scale matrices, respectively. Besides, dof parameters of prior state and likelihood all are Gamma distribution in IVBCKF. $\chi_{k}$ and $\phi_{k}$ denote shape parameter and rate parameter of dof parameters of prior state, respectively. ${ο}_{k}$ and $\varsigma_{k}$ denote shape parameter and rate parameter of dof parameters of likelihood, respectively [21]. Based on reference [21], the prior parameters are set as $\chi_{k}={ο}_{k}=5$, $\phi_{k}=\varsigma_{k}=1$, $u_{k}=8$, $t_{k}=5$. In order to obtain a balance between computational burden and accuracy, the starting particle number are set as $N_{start}=5000$. Besides, the maximum particle number must be less than starting number in MCUPF and the maximum particle number is set as $N_{max}=2000$. Following the advice of [4,24,35,36], the parameters of above filters are presented in Table 6. 

**Figure 7:**
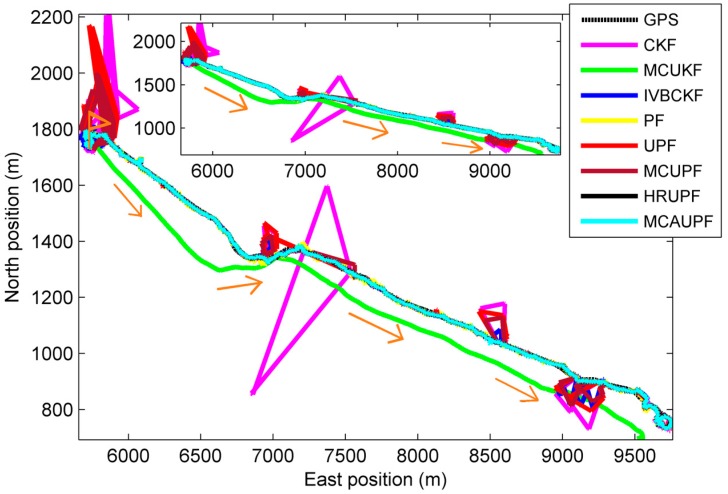
Paths taken by the proposed filter and existing filters.

**Figure 8:**
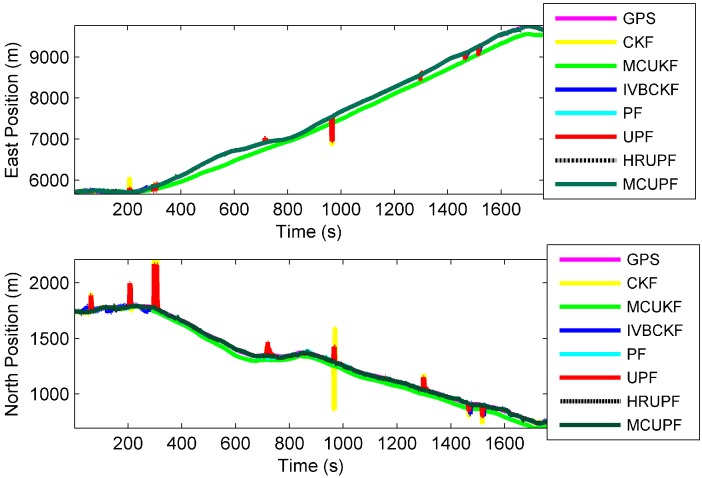
Position of AUV taken by the proposed filter and existing filters.

**Figure 9:**
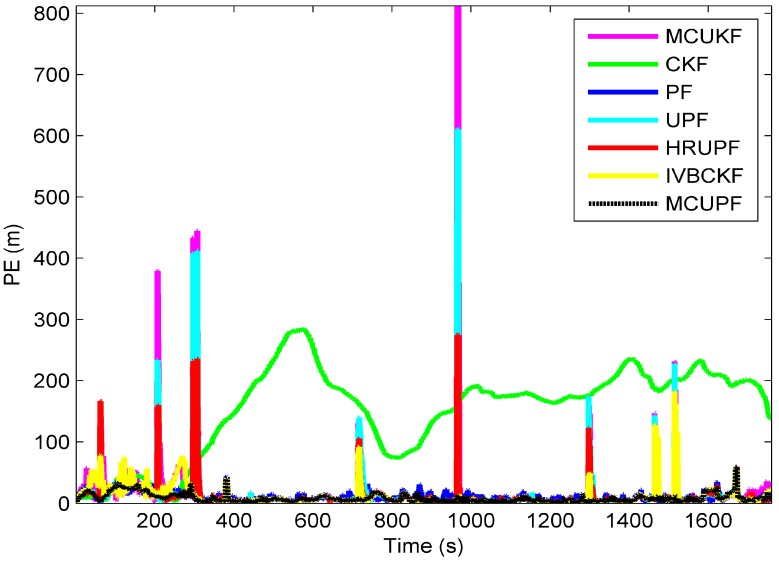
Position errors (PEs) of the proposed filter and existing filters in cooperative navigation (CN) of AUVs.

Experiment results are given as follows: Trajectory and positions taken by different filters are provided in Figure 7 and Figure 8, respectively, and APEs of different filters are shown in Figure 9, whose results and time consuming in a single step run are shown in Table 7. 

It can be seen from Figure 9 and Table 7 that all filters exhibit robustness in the presence of measurement outliers. Particularly, the proposed MCUPF has the best performance among these filters. Moreover, due to the use of the KLD-resampling method, the proposed MCUPF has lower computational cost as compared with HRUPF. 

### 4.2. Computational Complexity Analysis and Compares 

In this subsection, the computational complexity of CKF, MCUKF, PF, UPF, HRUPF, IVBCKF, and the proposed MCUPF in terms of the floating point operations are analyzed. Based on [38], the total floating point operations of these filters are as follows:

(47) $$S_{CKF}=O\left(2n^{3}+m^{3}\right)+2n\left(M_{f}+M_{h}\right)+6n^{3}+10nm^{2}+8mn^{2}+6n^{2}+2m^{2}+nm+m$$

(48) $$\begin{array}{ll} S_{MCUKF}= & O\left(6n^{3}+m^{3}\right)+\left(2n+1\right)\left(M_{f}+M_{h}\right)+6n^{3}+4nm^{2}+12mn^{2}\\ & +20mn+20n^{2}+4m^{2}+5m+8n \end{array}$$

(49) $$S_{PF}=O\left(n^{3}+N_{p}m^{3}\right)+\left(M_{f}+M_{h}\right)+N_{p}\left(2n^{2}+m\right)+S_{simple}$$

(50) $$S_{UPF}=N_{p}S_{HRUKF}+N_{p}\left[O\left(3n^{3}+2m^{3}\right)+\left(M_{f}+M_{h}\right)+8n^{2}+2m^{2}+2m+3n-3\right]+S_{sample}$$

(51) $$S_{HRUPF}=N_{p}S_{HRUKF}+N_{p}\left[O\left(3n^{3}+2m^{3}\right)+\left(M_{f}+M_{h}\right)+8n^{2}+2m^{2}+2m+3n-3\right]+S_{sample}$$

(52) $$\begin{array}{ll} S_{IVBCKF} & =O\left(n^{3}+T_{i1}n^{3}+m^{3}+3T_{i1}m^{3}\right)+\left[M_{f}+T_{i1}\left(2n+1\right)M_{h}\right]+3n^{2}\\ & +12T_{i1}nm^{2}+8T_{i1}mn^{2}+4T_{i1}n^{2}+6T_{i1}m^{2}+T_{i1}m \end{array}$$

(53) $$S_{MCUPF}=N_{p}S_{MCUKF}+N_{p}\left[O\left(3n^{3}+2m^{3}\right)+\left(M_{f}+M_{h}\right)+8n^{2}+2m^{2}+2m+3n-3\right]+S_{KLD}$$

where:

(54) $$\begin{array}{ll} S_{UKF} & =O\left(2n^{3}+m^{3}\right)+\left(2n+1\right)\left(M_{f}+M_{h}\right)+6n^{3}+10nm^{2}+8mn^{2}\\ & +\;13n^{2}+3m^{2}+7nm+4m+3n \end{array}$$

(55) $$\begin{array}{ll} S_{HRUKF} & =O\left(2n^{3}+2T_{i2}n^{3}+m^{3}\right)+\left(2n+1\right)\left(M_{f}+M_{h}\right)+6n^{3}+4nm^{2}+14mn^{2}\\ & +\left(15+8T_{i2}\right)n^{2}+8T_{i2}m^{2}+\left(7+16T_{i2}\right)nm+\left(4-3T_{i2}\right)n+\left(3-3T_{i2}\right)m \end{array}$$

where $S_{CKF}$, $S_{MCUKF}$, $S_{PF}$, $S_{UPF}$, $S_{HRUPF}$, $S_{IVBCKF}$, $S_{MCUPF}$, $S_{UKF}$, and $S_{HRUKF}$ denote the total floating point operations of CKF, MCUKF, PF, UPF, HRUPF, and IVBCKF, the proposed MCUPF, UKF, and Huber robust unscented Kalman filter(HRUKF), respectively; n and m denote the dimension of state vector and measurement vector, respectively; $M_{f}$ and $M_{h}$ denote the floating point operations of the computations of state equation and measurement equation, respectively; $N_{p}$ denotes particle number; $S_{simple}$ and $S_{KLD}$ denote the floating point operations of the simple-resampling and KLD-resampling, respectively; $T_{i1}$ and $T_{i2}$ denote iteration times of IVBCKF and HRUKF, respectively. In general, the iteration times of IVBCKF and HRUKF are more than 3 and the number of particles are tens of thousands. Thus, we can obtain the following equations based on (47)–(55):

(56) $$S_{IVBCKF}>S_{HRUKF}>S_{MCUKF}>S_{UKF}>S_{CKF}$$

(57) $$S_{HRUPF}>S_{MCUPF}>S_{UPF}>S_{PF}>S_{IVBCKF}>S_{MCUKF}>S_{CKF}.$$

It can be seen from (56) and (57) that four kind of PFs have higher computation complexity than others for using a large number of particles. Besides, the computation complexity of HRUPF is higher than the proposed MCUPF, due to the multi-iteration in HRUKF and the computation complexity of HRUKF is higher than the MCUKF. 

## 5. Conclusions

In this paper, a novel MCUPF is proposed for CN systems of AUVs with heavy-tailed measurement noises, where a MCC based robust UKF is used to generate the importance PDF and KLD measure is utilized to adjust the number of particles in the resampling process. Experimental results demonstrate that the proposed MCUPF has better estimation accuracy than existing state-of-the-art robust filters and lower computational complexity than existing improved PFs.

## Figures and Tables

**Table 1:** Algorithm of the standard unscented particle filter (UPF).

1. Initialization by Equations (5) and (6).
2. Importance sampling by Equations (7)–(21): Time update (Equations (7)–(10)); Measurement update (Equations (11)–(21)).
3. Resampling by Equation (22).
4. State estimation by Equations (23) and (24).

**Table 2:** Algorithm of the proposed robust UPF.

1. Initialization by Equations (5) and (6).	
2. Importance sampling like UPF (Equations (7)–(21)) but modify the Equation (14) as follows:	
$P_{zz,k|k-1}^{\left(j\right)}=\sum_{i=0}^{m}d_{i}\left(Z_{i,k|k-1}^{(j)*}-{\hat{z}}_{k|k-1}^{\left(j\right)}\right){\left(Z_{i,k|k-1}^{(j)*}-{\hat{z}}_{k|k-1}^{\left(j\right)}\right)}^{T}+{\tilde{R}}_{k}$	(39)
3. Resampling by Equation (22).	
4. State estimation by Equations (23) and (24).	

**Table 3:** Algorithm of the proposed maximum correntropy based unscented particle filter (MCUPF).

1. Initialization by Equations (6) and (7).	
2. Importance sampling as robust UPF	
3. Resampling: KLD-resampling as shown in Figure 1.	
$\left[\{{{\tilde{x}}_{k|k}^{\left(j\right)},{\tilde{f}}_{k}^{\left(j\right)}=\frac{1}{N_{KLD}}\}}_{j=1}^{N_{KLD}}\right]=\mathrm{KLD}-\mathrm{resampling}\left[\{{x_{k|k}^{\left(j\right)},f_{k}^{*(j)}\}}_{j=1}^{N}\right]$	(41)
4. State estimation by $N_{KLD}$ resampled particles from last step.	
${\hat{x}}_{k/k}=\sum_{j=1}^{N_{KLD}}{\tilde{f}}_{k}^{\left(j\right)}{\tilde{x}}_{k/k}^{\left(j\right)}$	(42)
${\hat{P}}_{k|k}=\sum_{j=1}^{N_{KLD}}{\tilde{f}}_{k}^{\left(j\right)}\left(x_{k|k}^{\left(j\right)}-{\hat{x}}_{k|k}\right){\left(x_{k|k}^{\left(j\right)}-{\hat{x}}_{k|k}\right)}^{T}$	(43)

**Table 4:** The parameters of employed sensors.

Sensors	Metric	Parameters
Acoustic modem: ATM-885	Working range Error rate	Up to 8 km Less than $10^{-7}$
GPS: OEMV-2RT-2	Position accuracy Date update rate	1.8 m (CEP) 10 Hz
DVL:DS-99	Working range Measurement accuracy	−150 m/s–200 m/s 0.1–0.3%
Magnetic Compass:H/H HZ001	Heading accuracy	0.3°

**Table 5:** The size of employed sensors.

Sensors	Size
Acoustic modem: ATM-885	140 mm in diameter 850 mm long
GPS: OEMV-2RT-2	160×160×160 mm
DVL:DS-99(Transceiver)	350×300×30 mm
Magnetic Compass:H/H HZ001	300×300×280 mm

**Table 6:** Parameters of employed filters.

Filters	Parameters
MCUKF	Kernel bandwidth σ=15
PF	The number of particles $N_{start}=5000$
UPF	The number of particles $N_{start}=5000$
HRUPF	Turning parameter γ=1.345 Iterated times m=3
IVBCKF	Prior parameters $\chi_{k}={ο}_{k}=5$, $\phi_{k}=\varsigma_{k}=1$, $u_{k}=8$, $t_{k}=5$
MCUPF	Kernel bandwidth σ=15 Bin size [10,10] Error bound ε=0.15 Bound parameter δ=0.01 Maximum particle number $N_{max}=2000$ Starting particle number $N_{start}=5000$

**Table 7:** Comparisons of averaged position errors (APEs) and computation time in a single step of the proposed filter and existing filters.

Filters	APE (m)	Time (s)
CKF [14]	152.0	$1.3644\times 10^{-4}$
MCUKF [34]	18.2	$1.6458\times 10^{-4}$
PF [14]	14.1	$2.6165\times 10^{-2}$
UPF [20]	15.1	0.1725
HRUPF [24]	12.4	0.3398
IVBCKF [21]	12.1	$7.9420\times 10^{-4}$
MCUPF	8.6	0.2156
